# Use of TikTok by nutrition healthcare professionals: analysis of the Italian context

**DOI:** 10.1093/eurpub/ckac131.333

**Published:** 2022-10-25

**Authors:** A Durbano, F Bert, A Pivi, G Lo Moro, G Scaioli, R Siliquini

**Affiliations:** 1 Department of Public Health Sciences, University of Turin, Turin, Italy; 2 A.O.U. City of Health and Science of Turin, Turin, Italy

## Abstract

**Background:**

Over the past 20 years, Social Media (SM) have become fundamental tools for quickly and effectively spreading health information in Europe. One of the most popular topics in the health sector is food and nutrition. TikTok is one of the most recent-born and most popular SM. Our study aims to analyze how healthcare professionals in the nutrition sector communicate through TikTok, and to assess characteristics of their accounts and videos.

**Methods:**

We included in the present study 53 Italian healthcare professionals who post videos on TikTok through a search on this SM using nutrition-related keywords. For each tiktoker, we described the characteristics of their last 10 videos, through an ad hoc checklist. We performed multilevel multivariable linear regression models in order to identify factors (healthcare professional or video related) that could be associated with a higher popularity of the video.

**Results:**

The 67.7% of the tiktokers considered were female; 46% had more than 30 years, 62.3% were biologists. The median number of likes was 300 (IQR 75 - 1070). The linguistic register was “formal” in the 11.3% of the videos. In the 31.9% of them, the location was the office of the healthcare professional. In the 67.3%, the topic was “diet-related”. Multilevel multivariable linear regression models showed that “diet-related” topics were associated with more likes (coeff 1111.63, p = 0.048), and comments (coeff 13.42, p = 0.018).

**Conclusions:**

Many Italian nutrition professionals are present on TikTok, and perform videos in their offices discussing mainly diet-related topics. There are no specific factors associated with the popularity of their videos, other than the presence of “diet-related” topics. Since the TikTok audience is very young, and wrong messages on this topic could lead to serious health-related consequences, there is the need to pay a specific attention on the contents of the videos, to avoid the spread of potentially dangerous information.

**Key messages:**

• Italian healthcare nutrition professionals are present on TikTok and perform videos with diet-related contents in their workplaces.

• Since wrong information on diet and nutrition could lead to serious health consequences for the audience, it is important to monitor the content of the videos to avoid the spread of dangerous messages.

